# Influence of the cutting speed in turning and force in diamond smoothing on the surface properties of pure nickel

**DOI:** 10.1038/s41598-026-48553-9

**Published:** 2026-04-12

**Authors:** Sepideh Ghorbanalipour, Hendrik Liborius, Jana Martini, Thomas Mehner, Andreas Nestler, Thomas Lampke, Andreas Schubert

**Affiliations:** 1 Chemnitz University of Technology, Micromanufacturing Technology, Chemnitz, Germany; 2 Chemnitz University of Technology, Materials and Surface Engineering, Chemnitz, Germany

**Keywords:** Diamond smoothing, Finishing, Nickel, Residual stress, Roughness, Turning, Engineering, Materials science

## Abstract

Nickel and its alloys offer excellent chemical resistance and mechanical properties under elevated temperatures. This makes them suitable for high- and low-temperature applications. Microreactors, due to their microscale dimensions, pose challenges for conventional joining techniques. Diffusion bonding offers a promising approach to overcome these challenges, with bond strength influenced by temperature, time, contact pressure, and surface state. This study investigates how face-turning and diamond smoothing affect the surface state of pure nickel. Specimens are face-turned at cutting speeds from 50 m/min to 400 m/min, followed by diamond smoothing with forces of 100 N to 200 N. Surface properties are characterized, and the results indicate that increasing the cutting speed decreases surface roughness values, coarsens crystallites, and increases tensile residual stresses. Smoothing at all forces induces compressive residual stresses and minimizes the surface roughness values at 100 N and 150 N. The findings highlight the influences of turning and diamond smoothing on the surface state of pure nickel, providing a basis for selecting surface preparation parameters that are expected to enhance diffusion bonding performance. With this knowledge, the field of application of diffusion bonding can be improved by achieving the suitable surface state.

## Introduction

Nickel and its alloys exhibit high strength, ductility, and resistance to corrosion and fatigue even at elevated temperatures, making them appropriate materials for demanding applications in harsh environments. However, nickel and its alloys are considered as difficult-to-machine materials due to their mechanical and thermal properties, including high work hardening and low thermal conductivity^1,2^, although pure nickel exhibits higher thermal conductivity than its alloys. Additionally, the small dimensions of microcomponents, e.g., found in microreactors, limit the use of conventional joining techniques. Diffusion bonding is an effective method for joining microstructured components with complex internal features, since no filler materials are needed. It is influenced by the joining temperature, time, and contact pressure. Furthermore, the microstructure of the surface layers and the state of the joining surfaces, including impurities and surface roughness, impact the bonding quality^3^. To achieve the desired surface properties (e.g., residual stresses, roughness values, microhardness, and microstructural features), machining techniques and surface modification methods, such as mechanical treatments, are applied^4,5^. Although the mentioned properties can be affected by the tool design and cutting parameters, mechanical and thermal loads, and their combination during machining impose limitations on achieving the desired surface state^6,7^. To overcome these restrictions, particularly in terms of surface roughness values and residual stresses, a subsequent mechanical treatment, such as diamond smoothing, may be implemented^8^.

Several studies have investigated the influence of machining parameters on the surface characteristics of Inconel 718. Frifita et al. (2020) demonstrated that surface roughness values decrease with increased cutting speed (*v*_c_) due to reduced burr formation. They reported that the feed (*f*) strongly influenced surface roughness values, where an increase in feed from 0.08 mm to 0.24 mm raised the values from 0.75 μm to 1.2 μm. Depth of cut (*a*_p_) showed an approximately linear effect on surface roughness values as higher feed or depth of cut increased the cross-section of the undeformed chip and hence deformed volume, resulting in higher surface roughness values. An increase in cutting speed also reduced the three components of the resultant force because higher speeds raised the temperature in the shear zone, which lowered the mechanical strength of the workpiece and reduced material hardness. This reduction was further supported by a lower friction coefficient at elevated temperatures. High feed and depth of cut corresponded to the higher cutting forces^9^. Similarly, Tan et al. (2022) reported that increased cutting speed tended to reduce the surface roughness values, whereas higher feeds increased the surface roughness values^10^. Zhou et al. (2014) investigated the subsurface microstructure and residual stresses in the machining of Inconel 718. The study showed that all components of the resultant force slightly decreased with increasing cutting speed (200 m/min – 350 m/min) due to thermal softening of the workpiece material. The surface layer exhibited a highly refined microstructure with nano-sized grains (50 –100 nm), significantly smaller than the bulk grains (5 –20 μm). That indicated that grain refinement occurred during surface formation under severe plastic deformation induced by the combined mechanical and thermal loads of high-speed machining. This refinement was attributed to the high dislocation density within the original grains, which promoted the subdivision of grains during deformation. Residual stresses were anisotropic, with more compressive stresses observed along the direction of feed motion compared to the cutting direction. Moreover, increasing cutting speed tended to shift stresses from compressive to tensile, primarily due to the rise in shear zone temperature. At higher speeds, the heat produced could not dissipate quickly enough, resulting in elevated temperatures^11^. Arunachalam et al. (2004) further found that increasing the cutting speed changed residual stresses from compressive to tensile and reduced surface roughness values due to rising temperatures and dominant thermal effects. A raise of the depth of cut increased both tensile residual stresses and surface roughness values^12^.

The influence of the tool geometry on residual stresses and surface roughness values when machining Inconel 718 has also been studied. Arunachalam et al. (2004) examined the effect of PCBN (polycrystalline cubic boron nitride) tool geometry on surface roughness when comparing round and square inserts. The application of round inserts resulted in much lower surface roughness values, for a feed of 0.15 mm and a depth of cut of 0.5 mm, *Ra* ≈ 0.4 μm, compared to square inserts, which led to values around 1.5 μm. The higher roughness values associated with square inserts were attributed to built-up edge formation and their smaller radius (corner radius 0.8 mm versus radius about 6 mm for round inserts), which theoretically led to higher surface roughness values^12^. Frifita et al. (2020) reported that a low corner radius (0.4 mm) resulted in lower components of the resultant force, compared to a larger corner radius (0.8 mm)^9^. Tool substrate and coating materials also affected both tool life and residual stresses. Zhou et al. (2014) found that ceramic tools (SiC whisker reinforced Al_2_O_3_) produced higher cutting forces compared to PCBN tools. This behavior is primarily attributed to the cutting edge radius, as the ceramic tool had an initial edge radius of 20 –25 μm, which increased due to wear during machining to about 50 μm along the cutting edge in the region where the surface was generated. In contrast, the PCBN tool showed a smaller initial edge radius of 10 –15 μm, which increased to around 20 μm during machining^11^. The larger cutting edge radius reduced the shear angle and increased the tool–workpiece contact area. As a result, the force components, particularly the passive component, became significantly higher^13^. In terms of residual stresses, whisker-reinforced ceramic tools generated much higher tensile stresses, mainly due to dominant thermal effects associated with their poor thermal conductivity, while PCBN tools resulted in more compressive surface residual stresses^11^. Arunachalam et al. (2004) highlighted that ceramic tools generally induced higher tensile residual stresses compared to PCBN tools in the machining of Inconel 718, primarily due to the poor thermal conductivity of mixed alumina ceramics and the restriction on coolant use, which intensified thermal effects and led to higher values of tensile residual stresses^12^. Additionally, Bushlya et al. (2012) compared uncoated and TiN-coated PCBN tools at cutting speeds of 250 m/min, 300 m/min, and 350 m/min. They noted that at 250 m/min, coated tools exhibited up to 20% longer tool life. However, this advantage decreased for cutting speeds above 300 m/min due to oxidation of the TiN coating at elevated temperatures. Tool wear was dominated by chemical and abrasive mechanisms in those cases^14^.

Diamond smoothing has been explored for its influence on the surface roughness and residual stresses. Nestler and Schubert (2015) examined the effects of slide diamond smoothing on aluminum matrix composites using monocrystalline spherical diamonds with radii of 1 mm, 2 mm, and 2.75 mm, while varying smoothing force (F = 50 N, 150 N, 250 N), speed (50 m/min, 100 m/min, 150 m/min), and feed (0.05 mm, 0.1 mm, 0.15 mm). They found that increasing the diamond sphere radius and decreasing the feed significantly reduces surface roughness values. The smoothing force had a noticeable influence on the results. By applying a force of 150 N, the smoothest surfaces and highest compressive residual stresses were observed. Lower force (50 N) left voids, while higher force (250 N) resulted in the lowest absolute residual stress values, likely due to material fatigue and surface scaling, which allowed for stress relaxation. Smoothing speed had minimal impact on the surface roughness values^8^. Similarly, Liborius et al. (2021) studied the effect of diamond smoothing on thermally sprayed (Al)CoCrFeNi(Mo) high-entropy alloy coatings. The process significantly reduced surface roughness values and led to increased surface hardness across all alloys, attributed to intense plastic deformation under higher normal forces compared to turning^15^. Maximov et al. (2022) examined diamond smoothing of AISI 304 stainless steel, noting that both diamond-sphere radius and smoothing force affected surface roughness values, with force having a stronger effect. The lowest surface roughness values were achieved using a sphere with the radius of 2 mm and forces between 400 N and 600 N. The diamond-sphere radius strongly influenced the microhardness, with 2 mm radius giving the highest microhardness, while 3 mm and 4 mm yielded lower microhardness values. Overall, a diamond sphere with a 2 mm radius combined with 600 N force provided both a low surface roughness value of 0.108 μm for *Ra* and a high hardness of 507 HV. Moreover, the feed strongly affected microhardness. Using a moderate feed of 0.07 mm simultaneously provided the minimum surface roughness value of approximately 0.09 μm and a microhardness of 415 HV due to the overlapping effect. Smoothing speed had minor impact on surface roughness value and microhardness, but speeds above 200 m/min were undesirable because they increased heat, accelerated diamond wear, and might damage the surface^16^.

Studies have demonstrated that surface state played a critical role in the mechanisms governing diffusion bonding and the resulting joint quality. Wei et al. showed that surface roughness strongly influenced interface contact, void formation, and bonding quality in diffusion-bonded 304 stainless steel. High roughness values resulted in large interfacial pores and unbonded regions due to limited asperity contact, whereas reducing roughness values increased the real contact area, enhanced asperity deformation, and promoted atomic diffusion. As a result, the interface bonding rate increased continuously with decreasing roughness values, reaching near-complete bonding at *Ra* ≈ 0.045 μm, demonstrating that surface roughness directly governed void closure and metallurgical bond formation^17^. Wu et al. demonstrated through modeling and validation that surface roughness strongly governed the dominant void-closure mechanisms during diffusion bonding. For smooth surfaces, void closure was mainly controlled by surface diffusion mechanisms, whereas increasing roughness values shifted the process toward creep- and grain-boundary-diffusion-dominated behavior due to reduced real contact area and larger voids formed by long-wavelength asperities. The study further showed that rougher surfaces introduced larger asperity wavelengths, which significantly affected contact geometry, void size, and diffusion pathways^18^.

Although there has been extensive research on machining and surface properties for nickel-based alloys and diamond smoothing for other materials, the impact of cutting and smoothing parameters on the surface state of pure nickel is still not well understood. The microstructure and physical characteristics of pure nickel can affect the responses of the material to machining and surface treatments. According to previous studies, cutting speed strongly influences the shear-zone temperature and cutting forces, which in turn affect the surface-layer properties and may alter the near-surface microstructure. Similarly, the applied force during diamond smoothing has been shown to govern the extent of plastic deformation and the generation of residual stresses. At the same time, it is not yet fully established which surface characteristics are most beneficial for the application of diffusion bonding of pure nickel. Therefore, this work investigates both turning and diamond smoothing in one study to provide complementary insights into how each process may differently modify the surface properties of pure nickel, with a particular focus on the influence of cutting speed in turning and applied force in diamond smoothing.

## Experiments

### Specimens

For the experimental investigations, circular disk specimens of pure nickel (Ni 201) with a diameter of 40 mm and a thickness of 5.2 mm were used. Moreover, each specimen is characterized by a central blind hole with a diameter of 8 mm to ensure constant cutting speed at the specimen surface while machining. According to the supplier’s material certificate, the chemical composition of the Ni 201 is given in Table 1, and the mechanical specifications are presented in Table 2.

**Table 1:** Chemical composition of Ni 201 according to the supplier’s material certificate.

Element	Mass fraction (%)
C	0.010
Si	0.08
Mn	0.07
S	0.001
Ti	0.002
Cu	0.008
Fe	0.19
Mg	0.011
Ni	99.53

**Table 2:** Mechanical properties of Ni 201 according to the supplier’s material certificate.

Yield strength (0.2% offset) (MPa)	Ultimate tensile strength (MPa)	Elongation at fracture (%)	Hardness (HB)
261	438	41	125

Before finishing, the specimens were heat-treated in a recirculating furnace under an air atmosphere. The heat treatment consisted of holding the specimens at 500 °C for one hour, followed by slow cooling inside the furnace. This enables to remove the work hardened layer resulting from pre-machining and ensures constant initial states for finish machining experiments. The material properties after this heat treatment were measured and summarized in Table 3. The crystallite size in this state is larger than the limit, which can be determined by XRD (≈ 300 nm).

**Table 3:** Measured material properties of the specimens after heat treatment.

Crystallite size (nm)	Microstrain (1°)	Microhardness (HV)	Principal stress (MPa)
> 300	0.111 ± 0.007	223 ± 21	-11 ± 2

### Tools

**Fig. 1:**
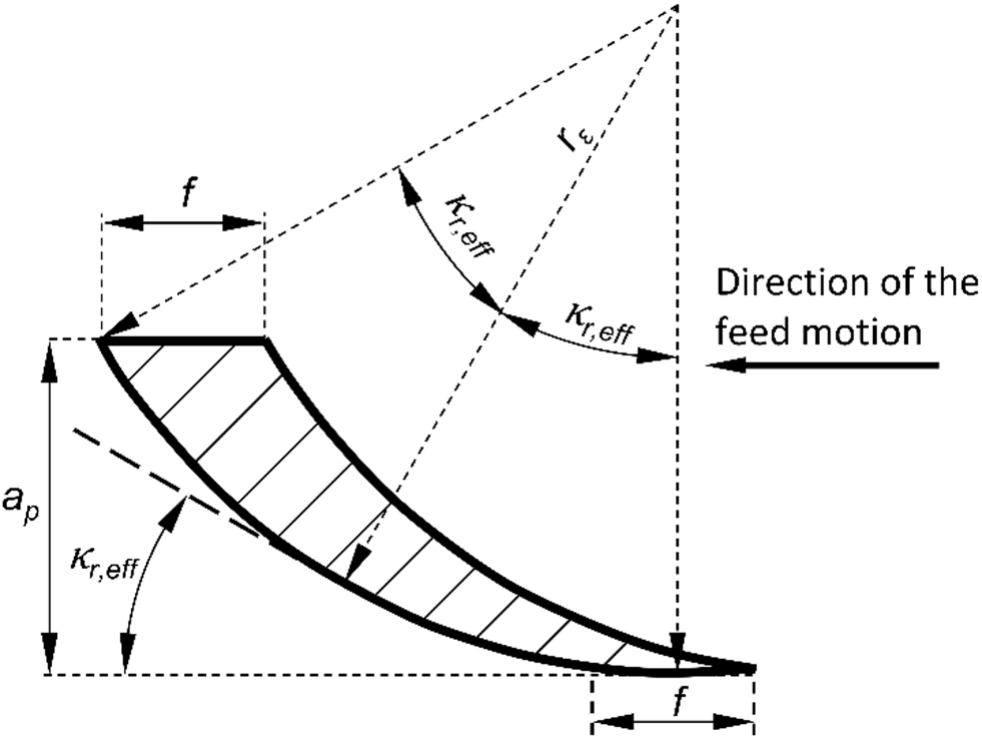
Schematic illustration of the cross-section of the undeformed chip showing the effective tool cutting-edge angle (a_p_: depth of cut, f: feed, κ_r, eff_: effective cutting-edge angle of the major cutting edge, r_ε_: corner radius).

Face turning was performed using PCBN-tipped indexable inserts. The cutting material consisted of 90% to 95% boron nitride particles with an average grain size of 1 μm and a cobalt binder. The tools, type CCGW 09T304 (SUMITOMO), featured a nominal rake angle of 0°, a clearance angle of 7°, and a sharp cutting edge (radius < 5 μm). The inserts were uncoated and mounted in a tool holder, type SCSCL 1212F09 (GARANT), providing a setting with a major cutting-edge angle of 45°. Considering the corner radius of the tool (*r*_ε_ = 0.4 mm) and the depth of cut of 0.2 mm, the effective cutting-edge angle of the major cutting edge (*κ*_r, eff_) differs from the nominal value. The effective angle can be determined geometrically, as shown in the schematic in Fig. 1, and represents the actual engagement angle of the major cutting edge with the workpiece during machining. For diamond smoothing, a tool with an MCD (monocrystalline diamond) spherical body exhibiting a radius of 2 mm was used. Each PCBN insert was used for a maximum of three experiments under identical cutting parameters. After machining, the tool wear was measured to ensure it remained within acceptable limits, with flank wear (*VB*) consistently between 30 μm and 40 μm. Similarly, the spherical tip of the diamond smoothing tool was inspected for wear to ensure that wear was minimal.

### Experimental investigations

In the experimental investigations, face turning was performed on a precision lathe, type PD 32 (SPINNER). The specimens were clamped on the outer diameter using a three-jaw self-centering chuck. All turning experiments were done coolant-free using a constant feed of 0.05 mm to minimize kinematic surface roughness and a depth of cut of 0.2 mm to remove a sufficient layer of material, including work hardened layer, for each pass, while the cutting speed was varied as listed in Table 4.

Diamond smoothing was performed on the specimens machined with a cutting speed of 200 m/min. During smoothing, the feed and speed were kept constant. The smoothing speed of 50 m/min was selected based on the tool supplier’s recommended operating conditions to ensure consistent performance and avoid tool damage. The feed of 0.025 mm was selected to achieve low surface roughness values combined with strong compressive residual stresses in the surface layer. Different smoothing forces according to Table 5 were applied. To improve the sliding behavior, emulsion flood cooling was used for smoothing. All experiments were performed three times.

After turning and diamond smoothing, all specimens were cleaned by rinsing with ethanol and drying with pressurized air to remove chips, emulsion, or other surface contaminants. Visual inspection confirmed that the surfaces were free of debris, ensuring that surface characterizations were not affected by residual contaminants.

In the face turning experiments, the tool holder was mounted on a three-axis dynamometer type 9257 A (Kistler), connected to a charge amplifier type 5070 A (Kistler). This setup enabled separate measurement of the cutting force *F*_c_, the feed force *F*_f_, and the passive force *F*_p_, with all signals recorded at a sampling rate of 1000 Hz.

**Table 4:** Machining parameters for face turning.

Cutting speed (m/min)	Feed (mm)	Depth of cut (mm)
50	0.05	0.2
100		
150		
200		
250		
300		
350		
400		

**Table 5:** Machining parameters for diamond smoothing.

Smoothing force (*N*)	Feed (mm)	Smoothing speed (m/min)
100	0.025	50
150		
200		

### Analyses of the specimens

**Fig. 2:**
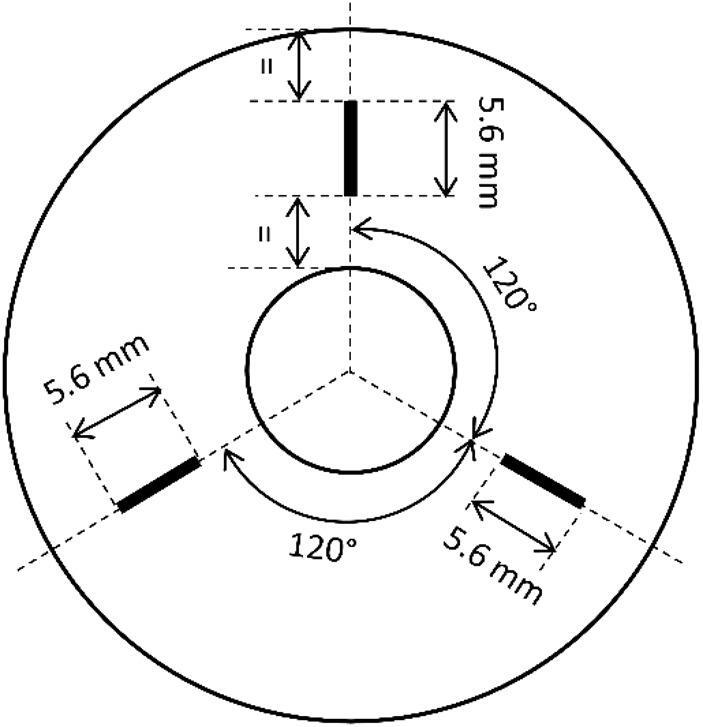
Schematic of the measurement positions for surface roughness by µCMM (Bruker alicona).

The geometrical properties of the machined surfaces were determined by an optical coordinate measuring machine µCMM (Bruker alicona) using the principle of focus variation. The size of the measuring field at each position (Fig. 2) was 0.5 mm × 6 mm. Before extracting the roughness profiles, the primary surfaces were adjusted by plane aligning using the subtraction method. The surface roughness of all specimens was determined in the direction of the feed motion along three lines on the specimen’s disk surface. These lines were oriented radially with an angular spacing of 120° between them, as shown in Fig. 2. The determination of the roughness values was done in accordance with ISO 4287. The evaluation length was 5.6 mm, and the distances from the inner hole and outer diameter were equal. The filtering of the profiles was done in accordance with ISO 16610-21 using a cut-off wavelength of 0.8 mm. The 3D measurement data was additionally used for the roughness determination in the cutting direction. Due to the size of the measuring fields, the evaluation length was limited to 0.5 mm, and it did not completely comply with current standards (only two single measured lengths for a cut-off wavelength of 0.25 mm). However, the measuring results enable the comparison of the influence of the different machining parameters. The profiles in the cutting direction were extracted in the valleys in the middle between the ridges of the feed marks. Because of the lower number of single measured lengths per roughness profile number of profiles per measuring field was increased to 15. For detailed analyses finish machined surfaces were also detected using a scanning electron microscope (SEM) type EVO 25 (Zeiss).

The Vickers hardness measurements were performed using the Tukon 1102 (Wilson Hardness) microhardness tester. A test force of 0.981 N was used (HV 0.1) with a test duration of 10 s. Ten individual measurements were carried out for each sample.

XRD residual-stress measurement was carried out using the sin²*ψ* method with a diffractometer D8 Discover (Bruker) and Co-Kα radiation (tube parameters: 35 kV, 40 mA, point focus). An area with a diameter of about 2.2 mm was irradiated. The measurements were conducted in three rotational directions (*ϕ* = 0°, 45°, 90°). The tilt angles (*ψ*) were selected in a way that there is an equal spacing of 0.1 (between 0.0 and 0.8) on a sin²*ψ* axis both in the positive and in the negative tilt direction. The {311} peaks of nickel were used for the measurements (elastic constants: Young’s modulus *E*^{311}^ = 203 GPa and Poisson’s ratio *ν*^{311}^ = 0.32). The software Leptos (Bruker) was applied in order to calculate residual stresses with a biaxial model with shear stresses. Error bars shown in the XRD-based results represent the fitting uncertainty related to the residual-stress determination using the sin²*ψ* method.

Crystallite sizes and microstrains were determined using the program Topas (Bruker) under consideration of device-related peak-broadening effects. The microstrain was calculated based on the full width at half maximum (FWHM) of the Gaussian component of the peak width. The value was obtained by dividing the FWHM (in degree) by the tangent of *θ* (tan(*θ*)), where *θ* is half of the diffraction angle. Similarly, the crystallite size was derived from the Lorentzian component of the peak width.

## Results and discussion

### Influence of cutting speed on components of the resultant force

**Fig. 3:**
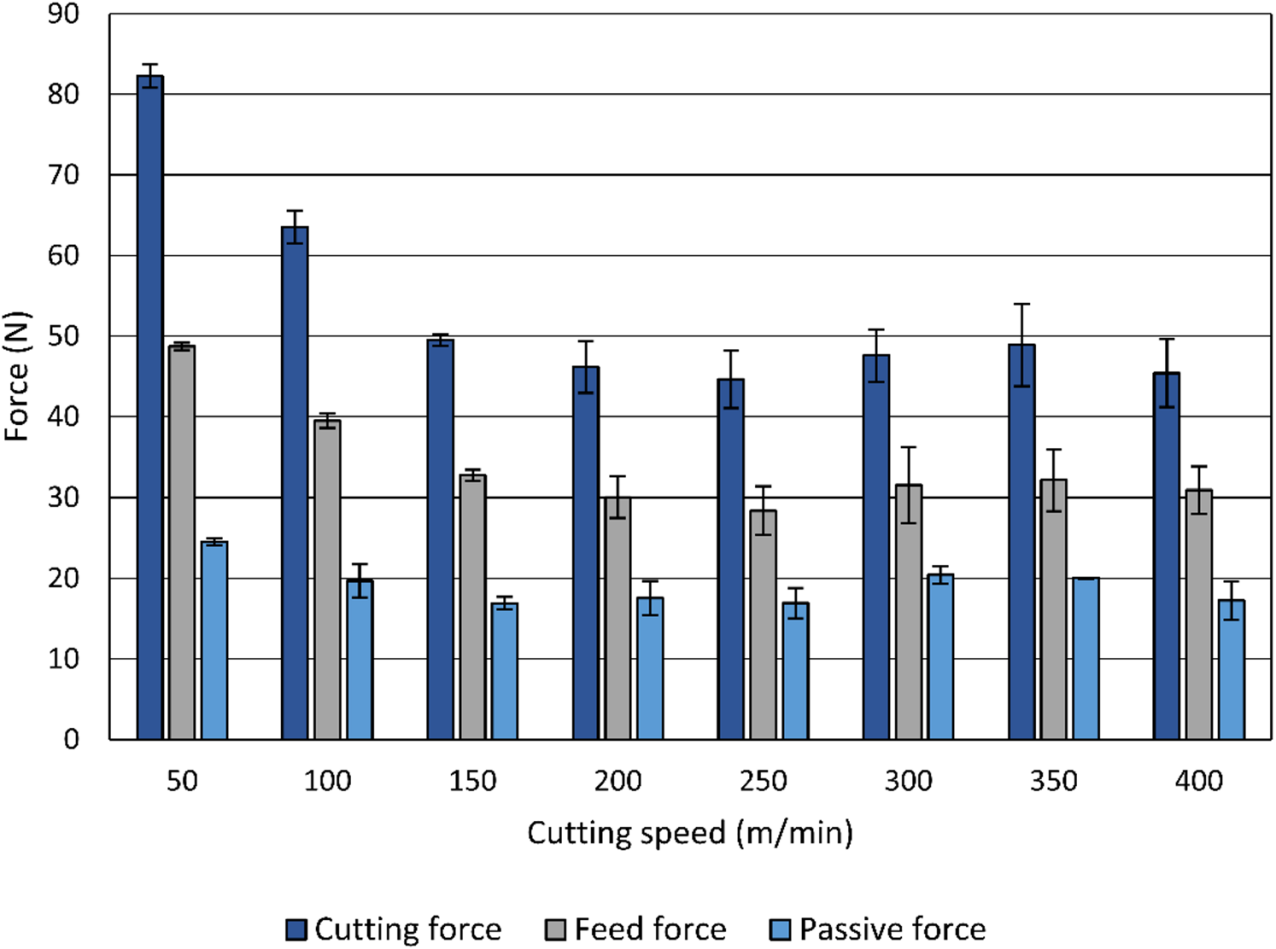
Influence of the cutting speed on the components of the resultant force.

The variation of cutting force, feed force, and passive force with cutting speed is illustrated in Fig. 3. For each specimen, cutting force signals were continuously recorded during face turning. The mean absolute value of each component of the resultant force was calculated over the steady-state portion of the cut, excluding entry and exit transitions. For each cutting condition, three specimens were machined, and the values presented in Fig. 3 correspond to the mean of the mean absolute forces from these three specimens, and the error bars represent the standard deviation of the three mean values, reflecting variability between experiments conducted under identical machining conditions. The cutting force is the largest component, starting at 82 N at 50 m/min and gradually decreasing to 46 N at 200 m/min, after which it stabilizes around 45 –49 N at higher speeds. The feed force shows a similar overall trend starting at 48 N at 50 m/min, dropping steadily to 30 N at 200 m/min, and remaining relatively consistent (31 –32 N) for higher speeds. The passive force remains the smallest component, ranging between 17 N and 25 N, with its maximum value occurring at 50 m/min.

**Fig. 4:**
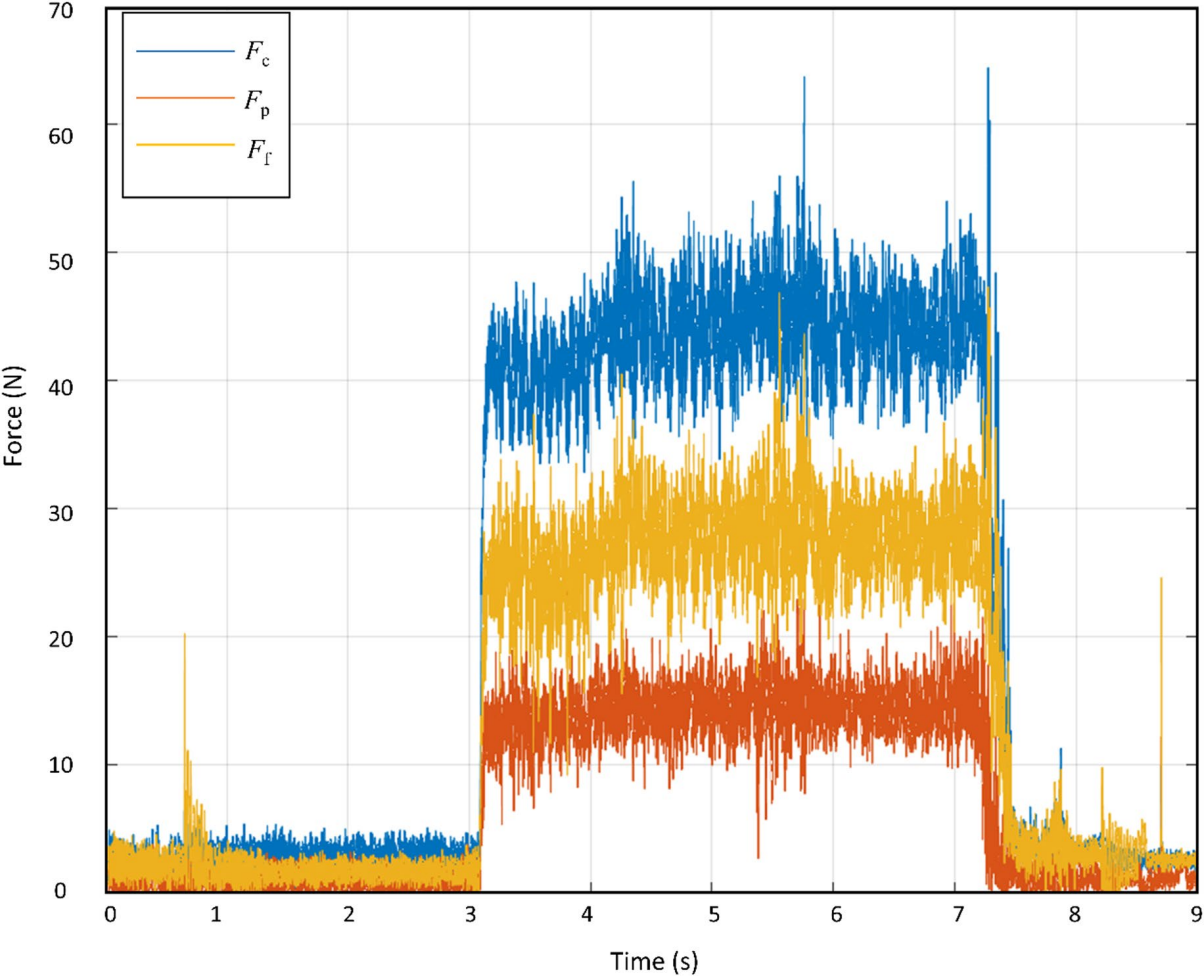
Profile of the components of the resultant force (a_p_ = 0.2 mm, f = 0.05 mm, v_c_ = 250 m/min), F_c_: cutting force, F_f_: feed force, F_p_: passive force.

To provide a clearer understanding of the force evolution during the cutting process, the force profile at a cutting speed of 250 m/min is shown in Fig. 4. The figure illustrates the temporal development of the cutting, feed, and passive force components, including tool entry, steady-state cutting, and tool exit. During the steady-state cutting interval, the force signals exhibit some fluctuations that are characteristic of continuous chip formation and stable turning of ductile metallic materials.

### Influence of cutting speed and smoothing force on geometrical surface properties

**Fig. 5:**
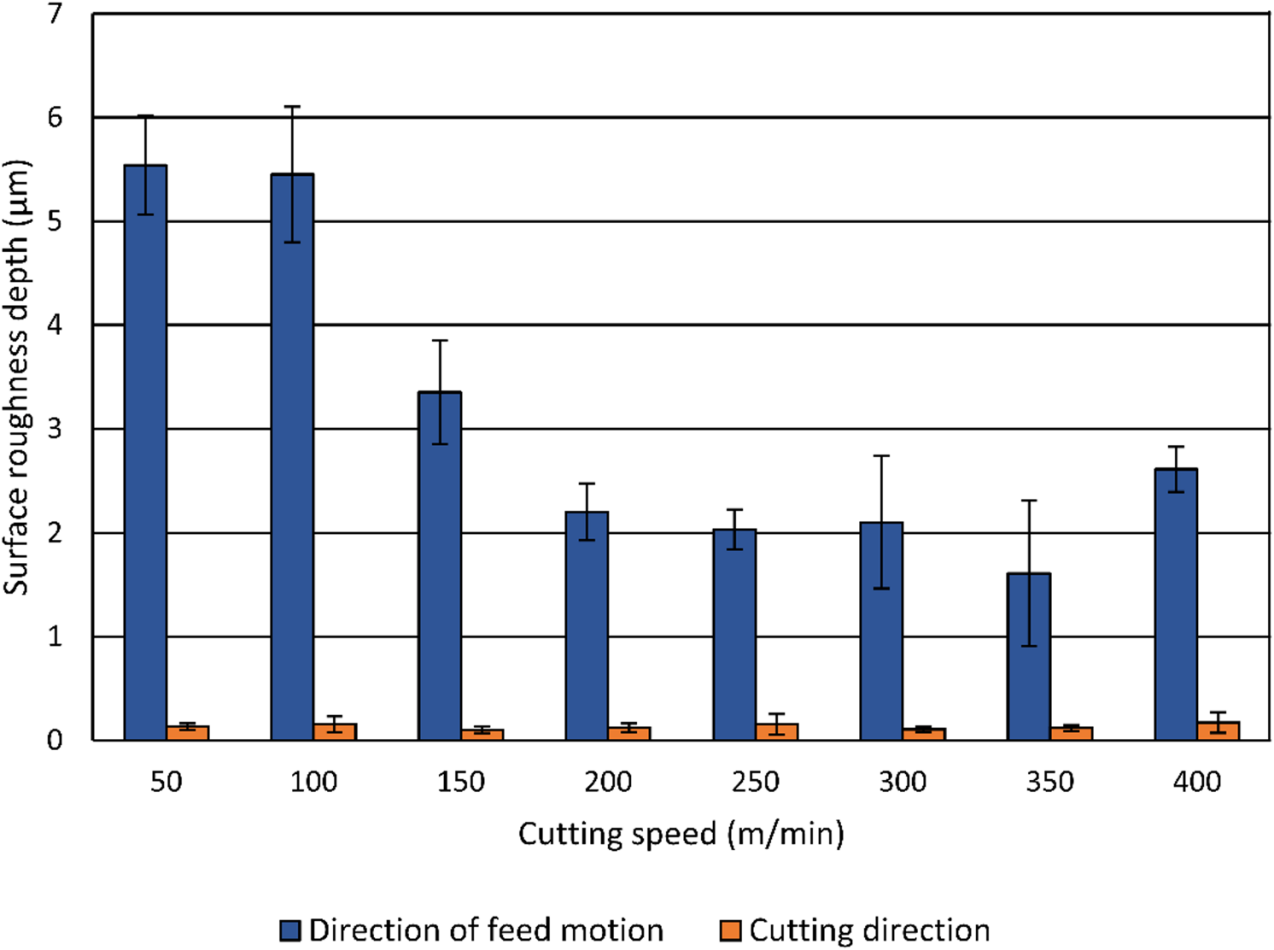
Influence of the cutting speed on the surface roughness depth.

The influence of the cutting speed on the surface roughness depth (*Rz*) in both feed and cutting directions is shown in Fig. 5. The surface roughness depth *Rz* is commonly used to evaluate the presence of surface asperities that may affect static or sliding contact performance. This makes *Rz* relevant for diffusion bonding applications, where the heights of the highest peaks and depths of the deepest valleys define the initial geometry of interfacial voids, and successful diffusion bonding requires the complete closure of these voids to achieve intimate atomic contact^19^. All measurements realized at specimens machined under the same conditions were used to calculate the mean values with standard deviations shown as error bars. The highest roughness values in the direction of feed motion were observed by machining at the lowest cutting speeds in the investigated range (50 m/min and 100 m/min), which also corresponds to the highest cutting and feed forces, as shown in Fig. 3. With increasing cutting speed, the surface roughness depth in the direction of feed motion decreases, reaching a minimum mean value of 1.6 μm at 350 m/min. The overall reduction in surface roughness values at higher cutting speeds is attributed to increased shear zone temperature and shear angle, which improve material removal. In contrast, the surface roughness depth in the cutting direction remains relatively constant across the investigated cutting speed range, between 0.102 μm and 0.172 μm, indicating that cutting speed predominantly affects the roughness profile in the direction of feed motion rather than the features in the cutting direction.

The surface roughness depth was significantly higher than the calculated kinematic surface roughness value of 0.78 μm (*f* = 0.05 mm) across all cutting speeds in the investigation range. To clarify this observation, the height profiles of the specimens turned at the cutting speed of 200 m/min were analyzed.

**Fig. 6:**
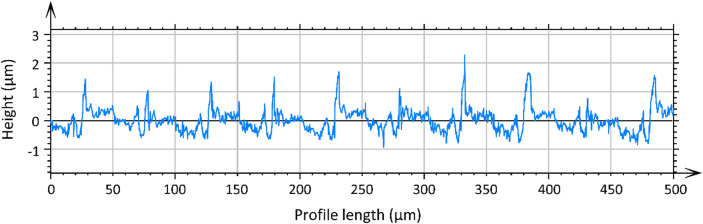
Height profile of a machined surface in the direction of feed motion for v_c_ = 200 m/min.

**Fig. 7:**
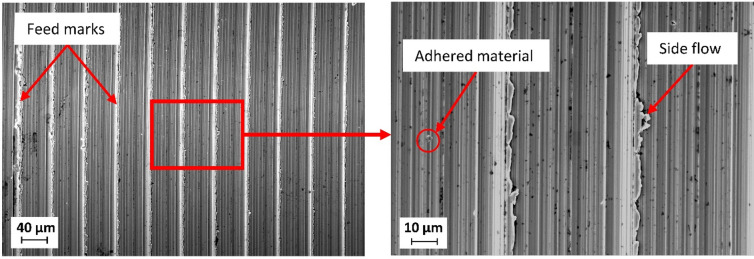
SEM micrographs of a machined surface for v_c_ = 200 m/min.

As shown in Fig. 6, the height profile of the sample at 200 m/min exhibits a periodic groove pattern originating from the tool feed marks, representing the kinematic contribution to surface roughness. Superimposed peaks with a height of up to ≈ 2.5 μm are also observed, corresponding to raised ridges and side flow along the feed marks, as illustrated in the SEM micrographs of the corresponding surface (Fig. 7). In addition, other surface imperfections, such as adhered material, are observed.

**Fig. 8:**
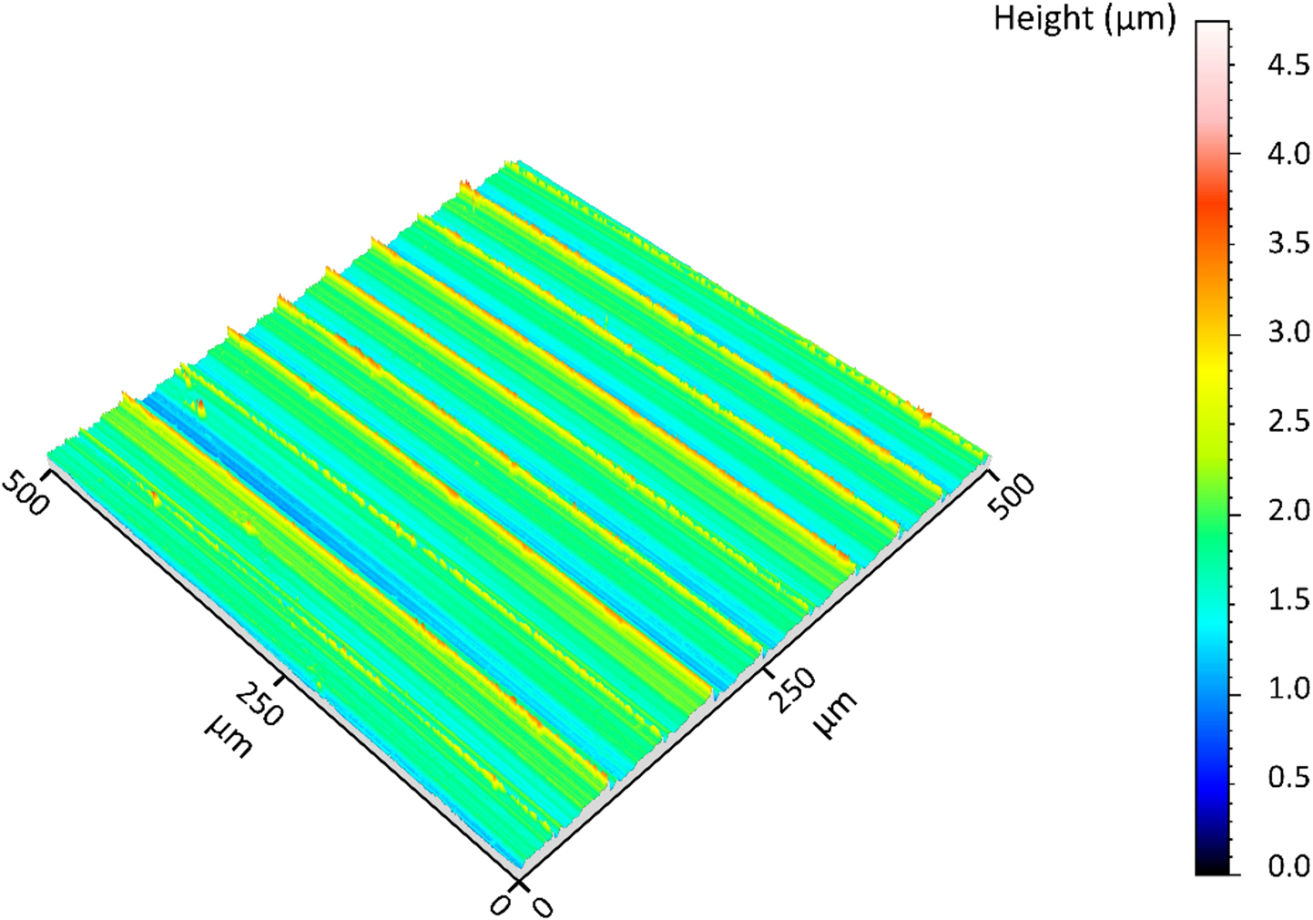
3D surface profile of a pre-machined specimen (a_p_ = 0.2 mm, f = 0.05 mm, *v*_c_ *= 200* m*/*min*)*.

Analyses of the results after diamond smoothing require sufficient information on the properties of the pre-machined surface. As the specimens were machined at a cutting speed of 200 m/min, the measured surface roughness depth values ranged from approximately 2 μm to 3 μm in the direction of feed motion and around 0.120 μm in the cutting direction. Figure 8 shows the three-dimensional surface profile of a specimen pre-machined for diamond smoothing, featuring pronounced feed marks with axial spacing consistent with the used feed of 0.05 mm.

**Fig. 9:**
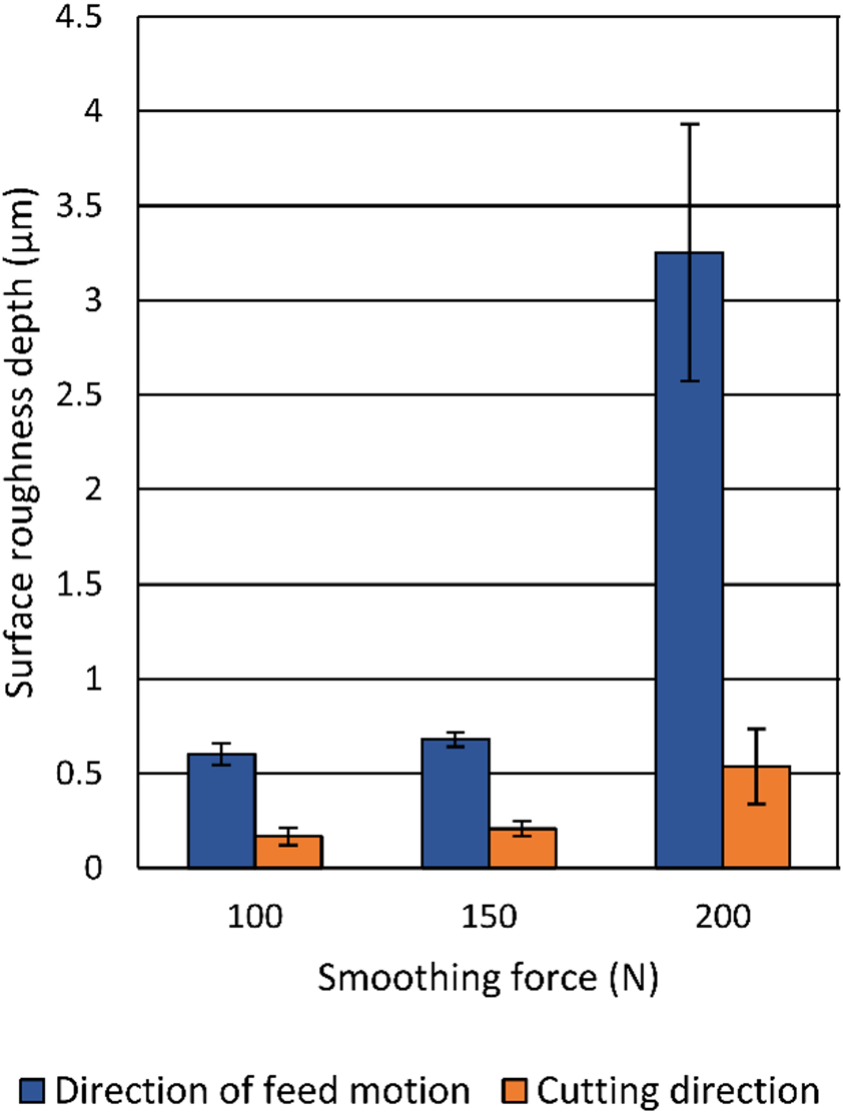
Influence of the smoothing force on the surface roughness depth.

**Fig. 10:**
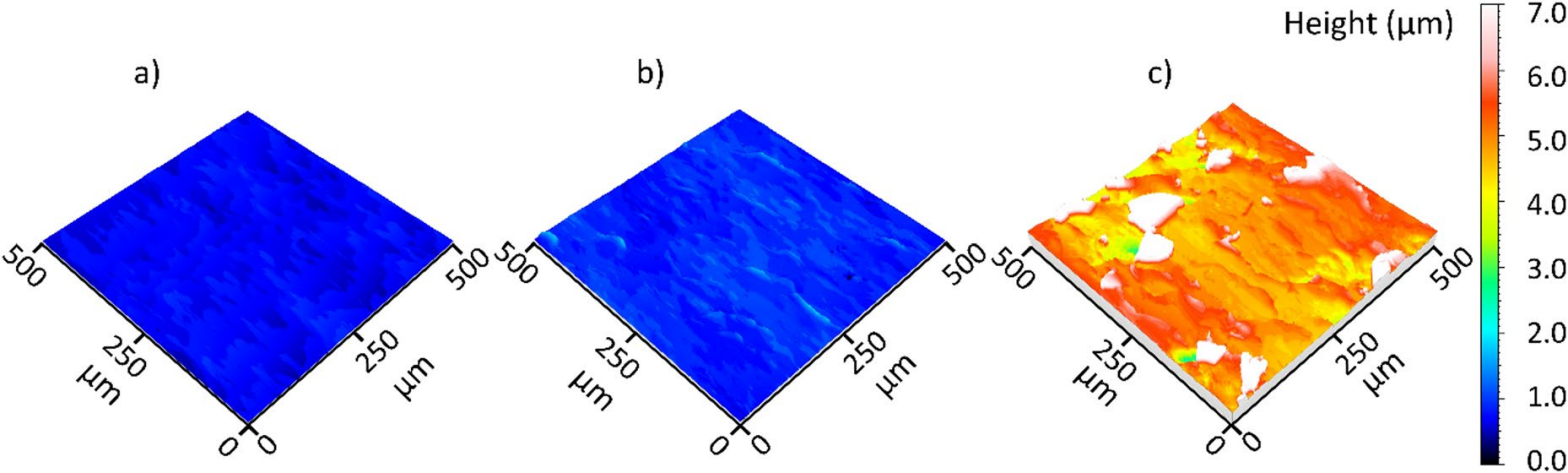
Influence of the smoothing force on the surface microstructure: (**a**) F = 100 N; (**b**) F = 150 N; (**c**) F = 200 N.

**Fig. 11:**
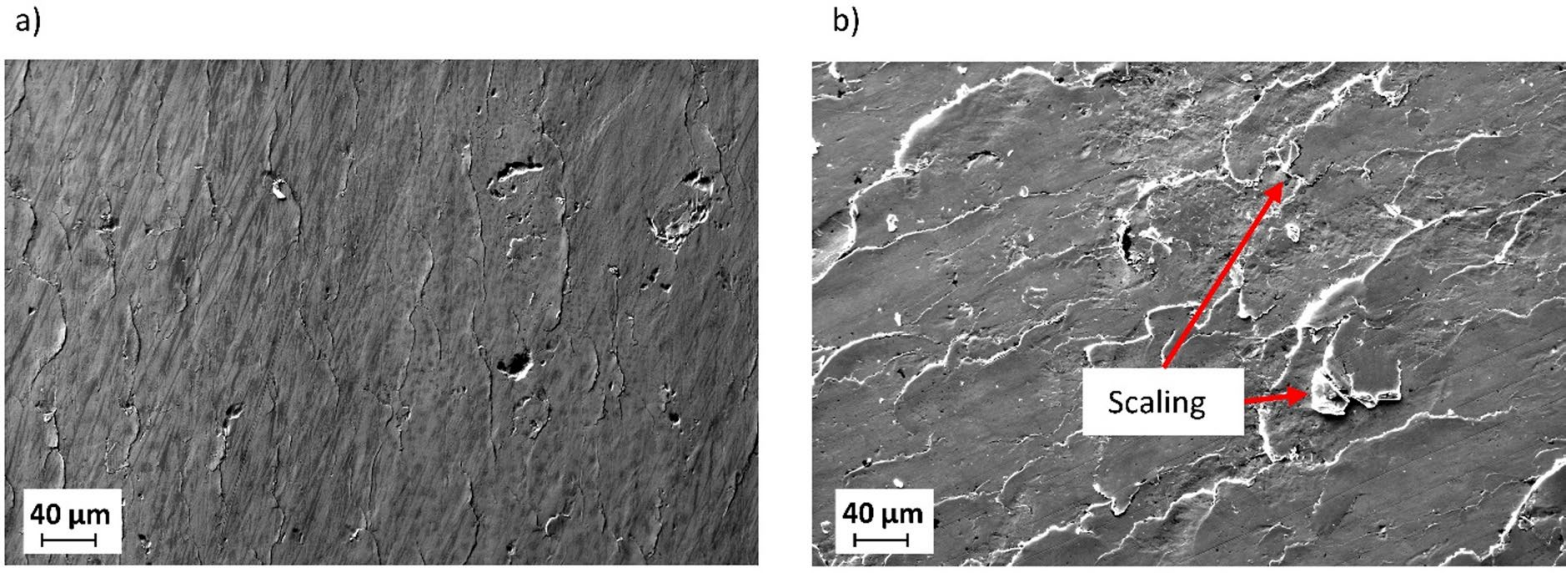
SEM micrographs of diamond-smoothed surfaces: (**a**) F = 150 N; (**b**) F = 200 N.

The application of diamond smoothing generally leads to a reduction in surface roughness depth. Figure 9 shows the effect of the smoothing force on surface roughness values in the direction of feed motion and in the smoothing direction, with mean values plotted and standard deviations represented by error bars. By applying smoothing forces of 100 N and 150 N, the lowest surface roughness values in the direction of feed motion in the tested range, approximately 0.6 μm to 0.7 μm, can be achieved, while the surface roughness values in the smoothing direction slightly increase to 0.168 μm and 0.208 μm at these stages. As can be seen in Fig. 10, these smoothing forces generated smooth surfaces with minimal surface imperfections, leading to reduced surface roughness values in the direction of feed motion. However, using a smoothing force of 200 N results in a significant increase in surface roughness values in both directions. SEM micrographs of the specimens smoothed under 150 N and 200 N (Fig. 11) highlight differences in their surface condition. Surface imperfections in the form of flake-like scaling with partially detached thin layers are more pronounced at the sample smoothed with a force of 200 N. These imperfections arise during smoothing because the tool’s pressure plastically deforms the material, causing material flow and shift ahead of the tool. Moreover, high force and the resulting contact pressure lead to a high degree of deformation, causing fragments of the surface layer to detach as scaling and subsequent stress relaxation^8,20^.

### Influence of cutting speed and smoothing force on surface-layer properties

**Fig. 12:**
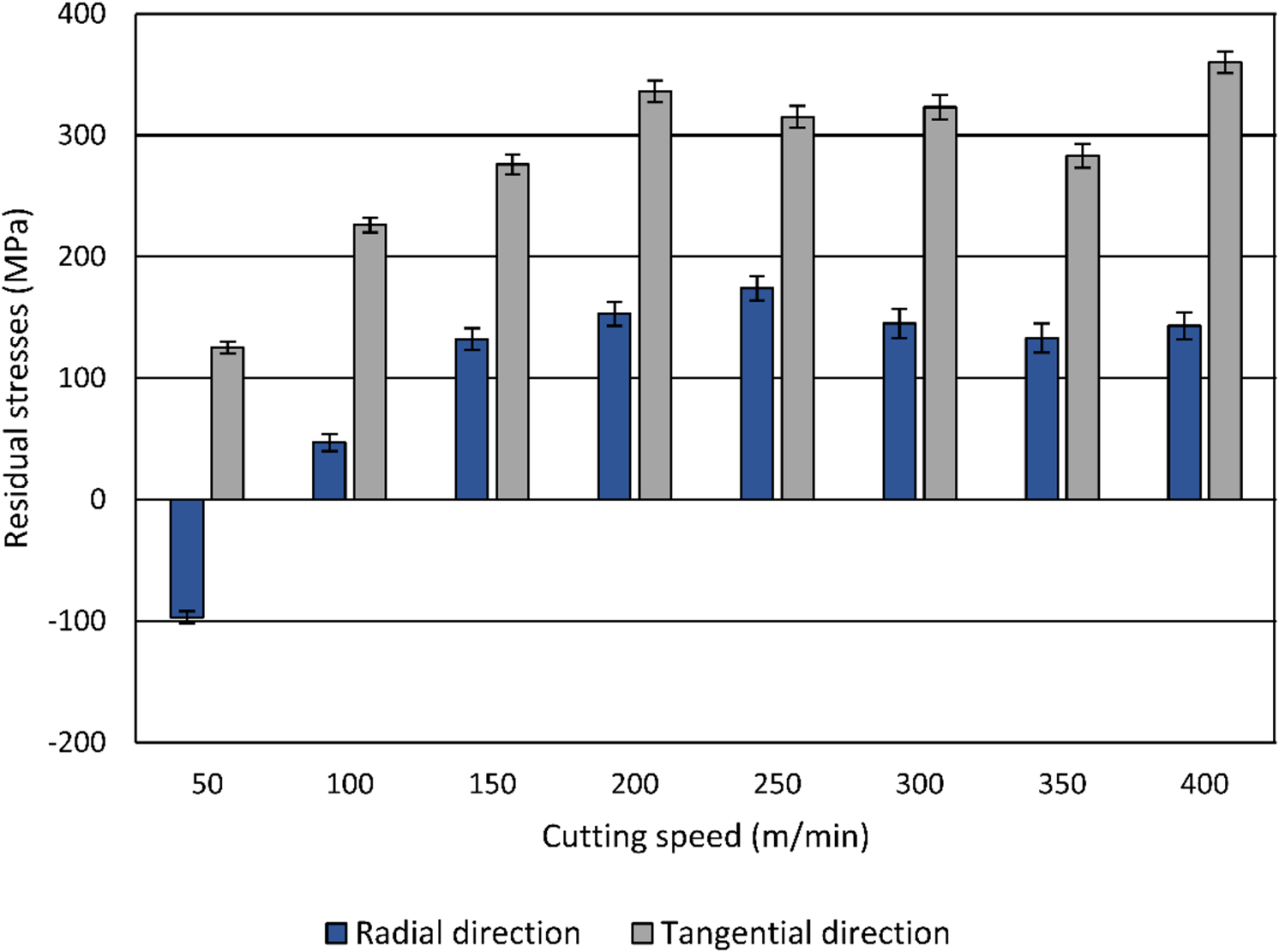
Influence of the cutting speed on the resulting residual stresses.

Cutting processes have been shown to alter the surface layer’s microstructure, leading to changes in residual-stress profiles in the workpiece material as a result of the combined effects of localized temperature and mechanical loads during machining^21^. Elevated localized heat generation during machining often leads to the formation of near-surface tensile residual stresses^2^. The influence of the cutting speed on the resulting residual stresses is shown in Fig. 12.

Residual stresses were measured radially in the direction of feed motion (*σ*_rs, r_) and tangentially in the cutting direction (*σ*_rs, t_). After heat treatment and prior to turning, the surface layer of the specimens was almost free of residual stresses. In the radial direction, residual stresses increase significantly with rising cutting speed, shifting from compressive residual stress at 50 m/min (approximately − 100 MPa) to tensile residual-stress values above 100 MPa from 150 m/min onward. The increase appears to plateau at higher cutting speeds. In the tangential direction, residual stresses remain consistently tensile across all cutting speeds, showing a general upward trend, peaking at around 360 MPa at 400 m/min. These results indicate that higher cutting speeds lead to increased tensile residual stresses, particularly in the tangential direction, which is attributed to a higher shear-zone temperature. Moreover, when machining at low cutting speeds, the compressive residual stresses in the radial direction indicate the dominance of plastic deformation.

**Fig. 13:**
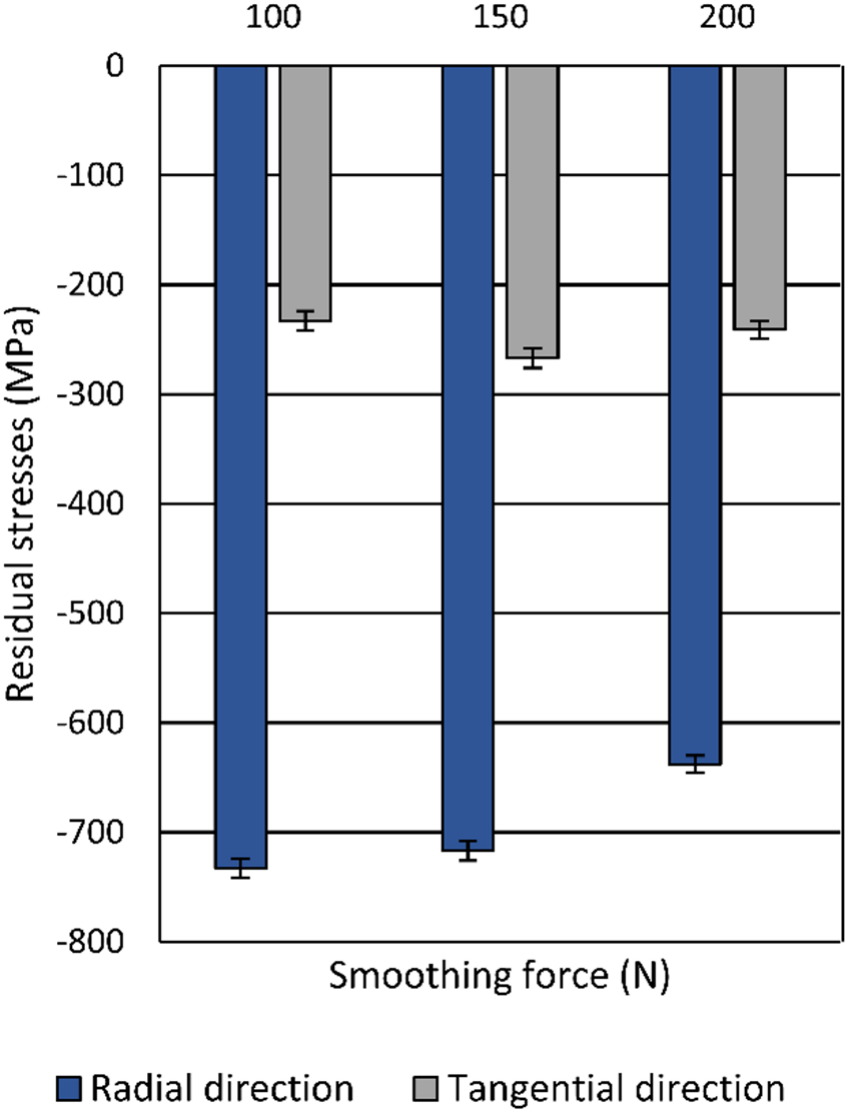
Influence of the smoothing force on the resulting residual stresses.

Regardless of the applied smoothing force, diamond smoothing leads to compressive residual stresses both in the radial and the tangential direction. Figure 13 illustrates the effect of the smoothing force on surface residual stresses. All measured values are negative, indicating that the residual stresses are compressive, while the absolute values in the radial direction are higher than those in the tangential direction. Considering the residual-stress state in the surface layer of a pre-machined specimen (*σ*_rs, r_ ≈ 150 MPa, *σ*_rs, t_ ≈ 330 MPa), diamond smoothing leads to a significant increase in the absolute values of residual stresses in the radial direction, whereas in the tangential direction, the absolute values were reduced across all investigated smoothing forces. The highest absolute value of compressive residual stresses in the radial direction of approximately − 740 MPa was reached at a smoothing force of 100 N. Its absolute value exceeds the ultimate tensile strength of the bulk material listed in Table 2, indicating a pronounced work-hardening effect. With increasing force, the compressive residual stress in the radial direction gradually decreased to around − 630 MPa at 200 N. In the tangential direction, the residual stresses ranged from − 240 MPa to -270 MPa, with the maximum absolute value at a smoothing force of 150 N. These results suggest that higher smoothing forces do not necessarily lead to greater compressive residual stresses, possibly due to residual stress relaxation caused by scaling occurring at elevated forces, as shown in Fig. 11.

**Fig. 14:**
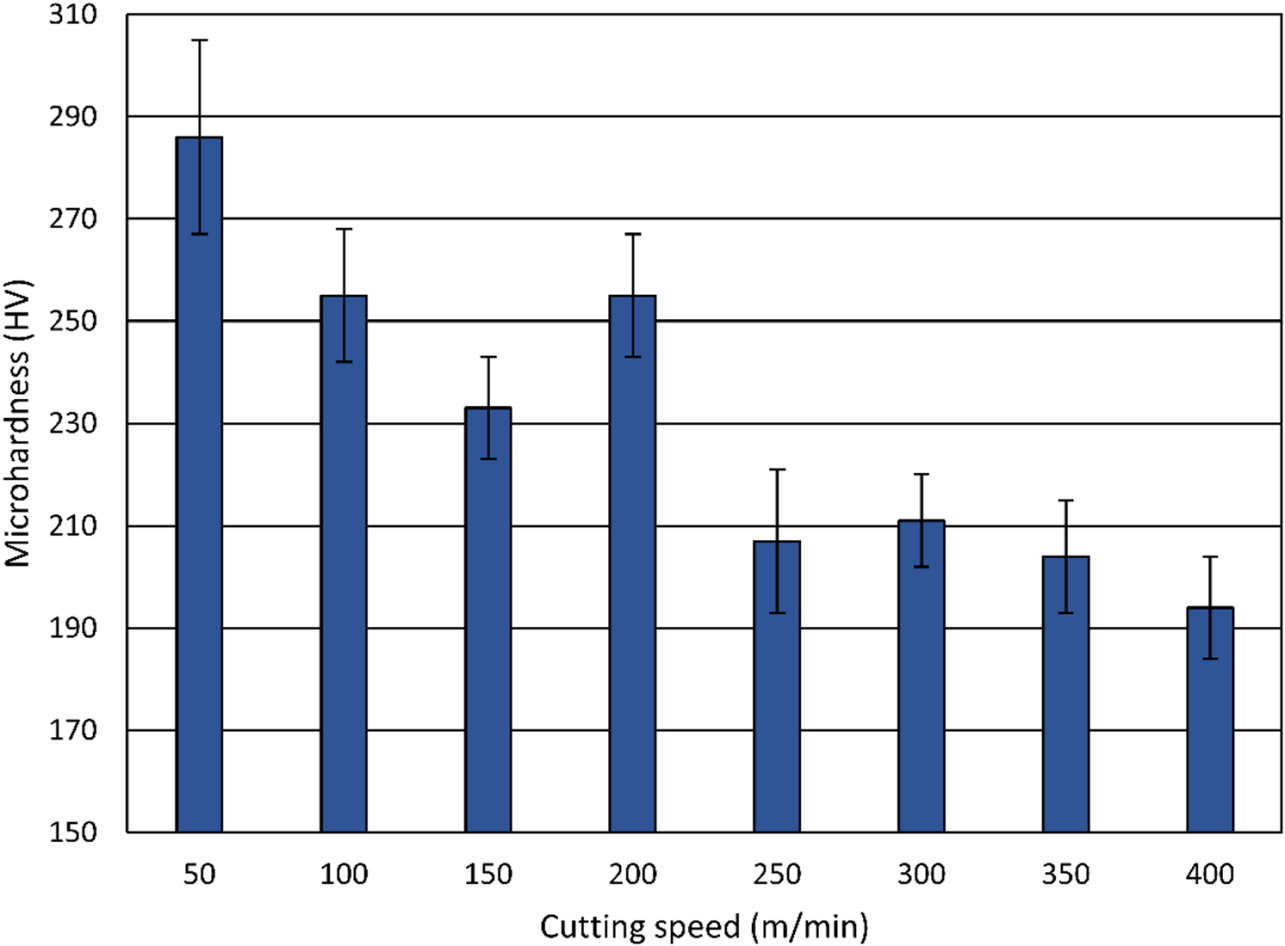
Influence of the cutting speed on the microhardness.

The variation of surface microhardness with cutting speed is illustrated in Fig. 14. The plotted values represent the mean value of repeated measurements on specimens, with standard deviations displayed as error bars. The initial microhardness of the heat-treated, unmachined specimens is 223 HV ± 21 HV, where the measured value may be influenced by the indentation size effect. After turning, the surface microhardness values increase at cutting speeds up to 200 m/min, with the highest microhardness value (≈ 285 HV) at the lowest cutting speed of 50 m/min, indicating that a slower cutting speed promotes higher work hardening of the surface layer. For cutting speeds beyond 200 m/min, the surface microhardness decreases, reaching its lowest value (≈ 195 HV) at 400 m/min.

**Fig. 15:**
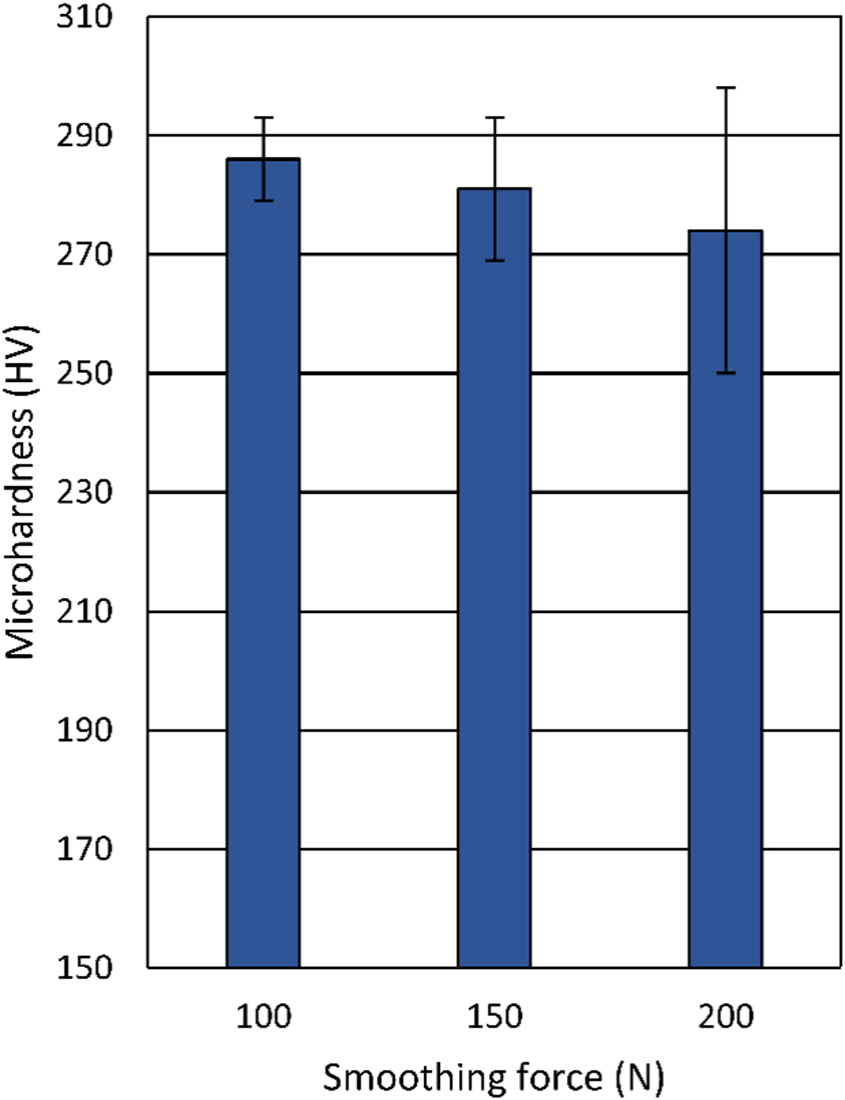
Influence of the smoothing force on the microhardness.

The effect of the applied force on the microhardness of the samples after diamond smoothing is shown in Fig. 15. The values correspond to the mean values of 10 measurements, with error bars indicating standard deviations. At all forces analyzed (100 –200 N), the smoothed surfaces exhibit higher hardness values (274 HV – 286 HV) compared to the corresponding turned surface (≈ 255 HV). This increase clearly demonstrates the strengthening effect of diamond smoothing, which introduces work hardening as a result of severe plastic deformation. However, a slight decrease in hardness is observed with increasing smoothing force, from 286 HV at 100 N to 274 HV at 200 N.

**Fig. 16:**
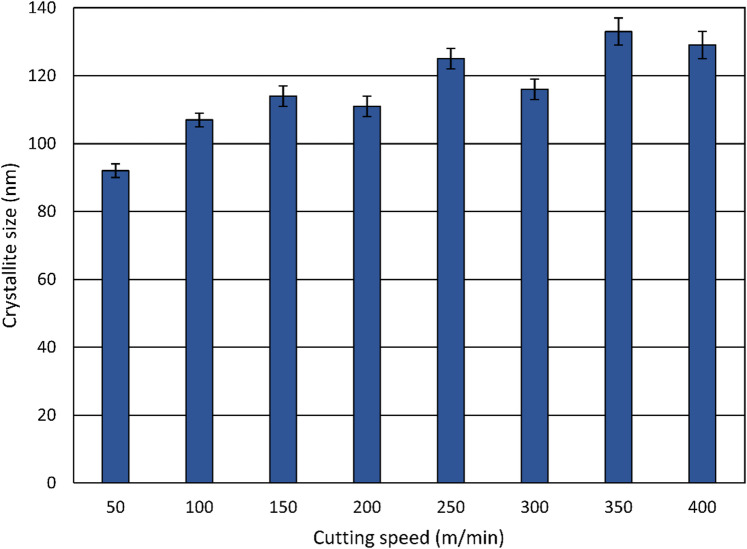
Influence of the cutting speed on the crystallite size.

The crystallite size after heat treatment was larger than 300 nm according to Table 3, and it is clearly influenced by the turning process, as shown in Fig. 16. For all cutting speeds investigated, turning leads to a refinement of the crystallites, reducing the crystallite size to below 140 nm. This reduction is mainly caused by the severe plastic deformation in the tertiary shear zone, which applies high plastic strain and subdivides the grains, leading to the refined microstructure after turning^22^. The crystallite size decreases to about 90 nm at 50 m/min. However, at higher cutting speeds, the degree of refinement is less pronounced, reaching around 130 nm at 400 m/min.

**Fig. 17:**
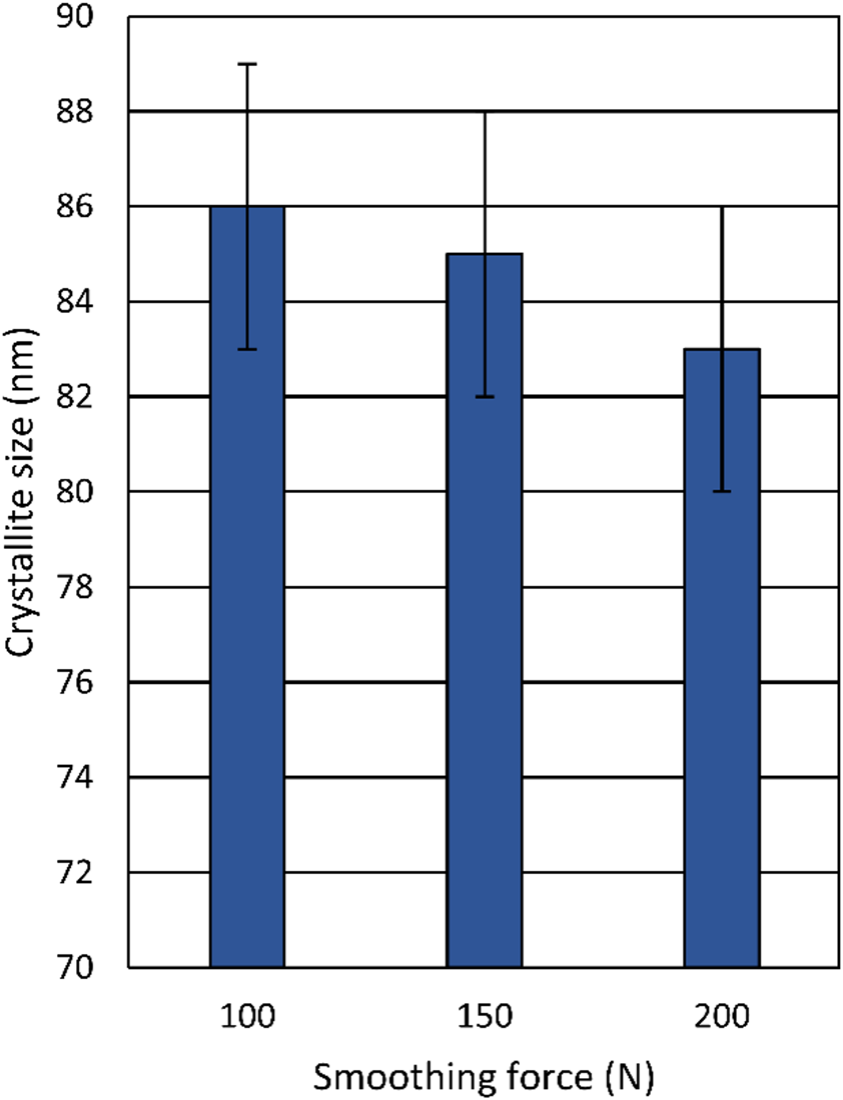
Influence of the smoothing force on the crystallite size.

As shown in Fig. 17, the crystallite size further decreased to 86 nm after the diamond smoothing process, compared to about 110 nm after turning with a cutting speed of 200 m/min. Moreover, by applying a higher smoothing force, the crystallite size decreases to 83 nm at the smoothing force of 200 N. This crystallite refinement is attributed to plastic deformation mechanisms, specifically the accumulation of dislocations within the grains, which form low-angle grain boundaries in the surface layer, subdivide the grains into smaller subgrains, and reduce the overall crystallite size^23^.

**Fig. 18:**
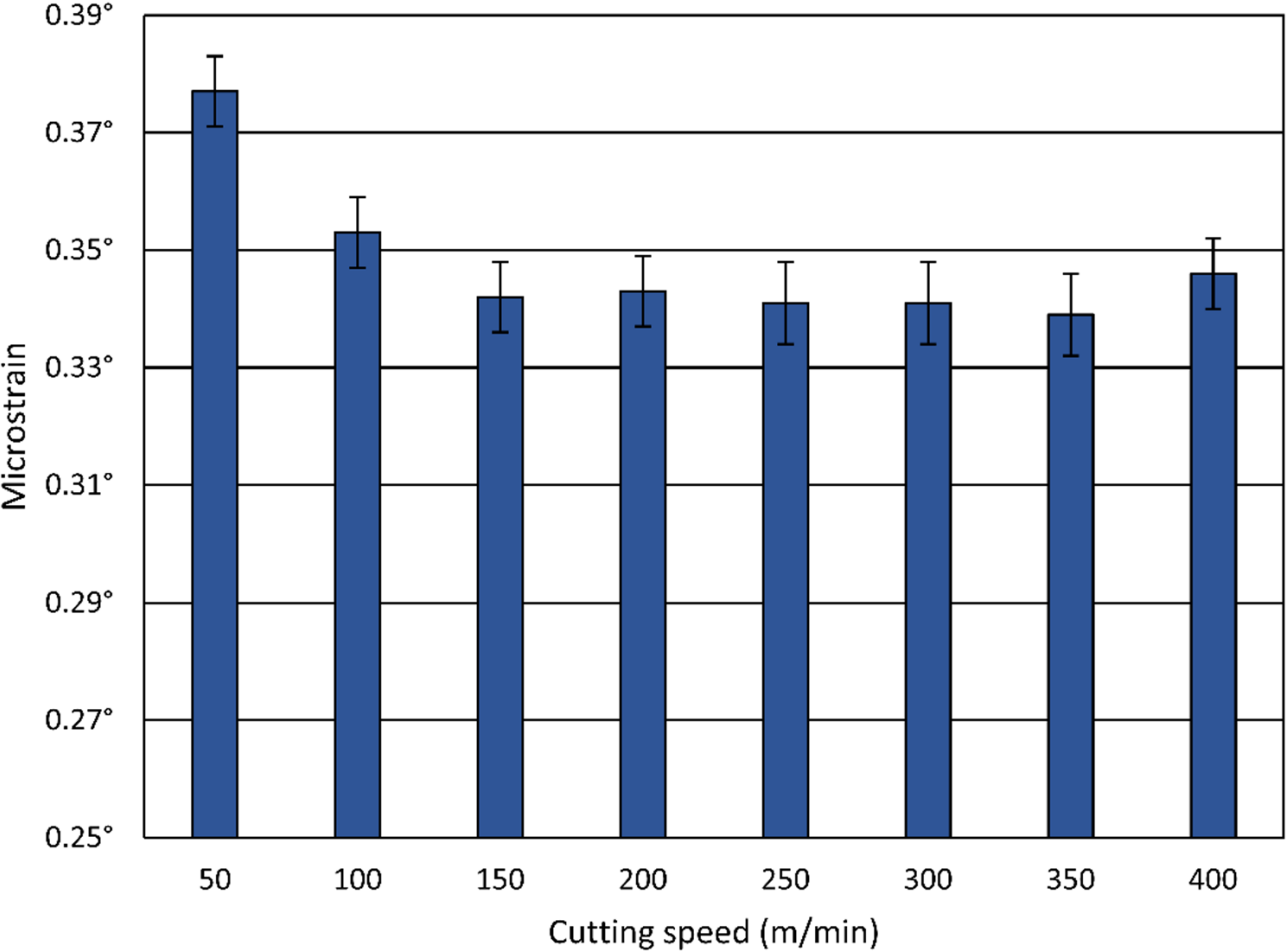
Influence of the cutting speed on the microstrain.

Figure 18, based on XRD analysis, shows that the microstrain decreases with increasing cutting speed. The highest microstrain was observed at the lowest cutting speed of 50 m/min, reaching about 0.375°. As the cutting speed increases, microstrain values decline and stabilize around 0.34° from 150 m/min onward. The decreasing microstrain with increasing cutting speed is attributed to thermal softening effects, which reduce the extent of plastic deformation and lower the dislocation density in the surface layer. Compared to the initial microstrain of approximately 0.111° after the heat treatment, i.e., before turning, the overall increase after machining highlights the effect of severe plastic deformation during the cutting process^24^.

**Fig. 19:**
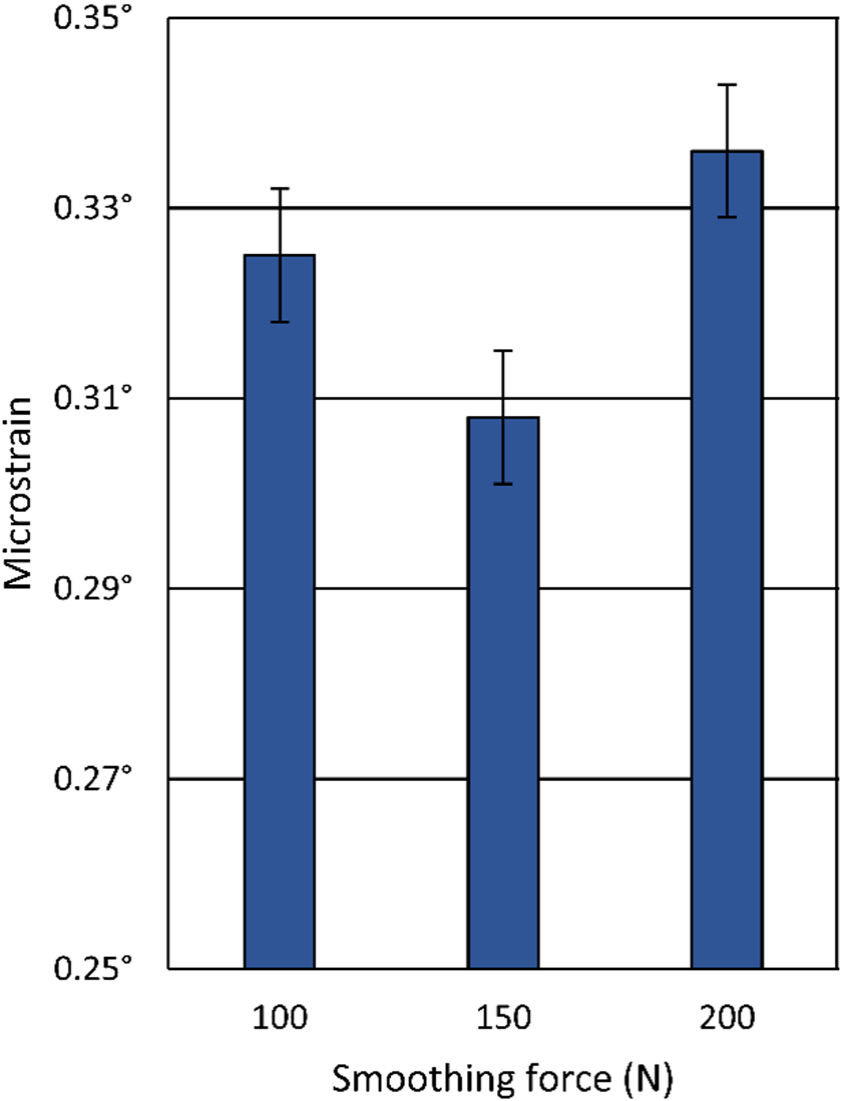
The influence of the smoothing force on the microstrain.

The microstrain after diamond smoothing decreased slightly, as it was 0.343° before it, and the mean values remained below 0.336° as illustrated in Fig. 19, with only minor variations observed and no clear trend with changes in the smoothing force. Error bars represent XRD fitting and measurement uncertainty.

### Correlation of surface roughness values and microstructure results

At low cutting speeds in the investigated range, high surface roughness values, elevated microhardness, higher microstrain values, crystallite refinement to about 90 nm, and compressive residual stresses in the radial direction at the lowest cutting speed (50 m/min) are observed. These conditions are associated with severe plastic deformation. As the cutting speed increases, the reduction in cutting forces and smoother surface finish coincide with lower microhardness, reduced microstrain, and reduced degree of crystallite refinement, indicating the growing influence of thermal softening. Thus, the interplay between mechanical loads and thermal effects during machining directly influences the surface layer properties, including microhardness, residual stresses, microstrain, and crystallite size.

Moreover, the results indicate a correlation between surface roughness values, residual stress state, and crystallite refinement during diamond smoothing. Smoothing forces of 100 –150 N resulted in the lowest surface roughness values in the tested range and strong compressive residual stresses. These conditions also coincide with the maximum surface microhardness and a notable reduction in crystallite size to below 90 nm. At excessive force (200 N), surface roughness values increased, and the absolute values of the compressive residual stresses decreased slightly, indicating local relaxation and the generation of surface imperfections.

## Summary and conclusions

The experimental results indicate that at low cutting speeds, surface state is dominated by mechanical effects, resulting in elevated cutting forces, a pronounced roughness in the direction of feed motion, high microhardness, increased microstrain, strong crystallite refinement, and partially compressive residual stresses. With increasing cutting speed, reduced forces, and improved surface finish are accompanied by diminished work hardening, lower microstrain, reduced crystallite refinement, and a transition toward tensile residual stresses, indicating the growing influence of thermal softening. These findings highlight that surface roughness values in the direction of feed motion are strongly coupled to subsurface deformation mechanisms, whereas roughness values in the cutting direction remain relatively constant. Under the given circumstances and only concerning the geometrical surface properties, a cutting speed of at least 150 m/min is necessary, since lower cutting speeds within the investigated range result in surface roughness depth values up to 5.5 μm, which are significantly higher than the theoretical value of 0.78 μm. However, depending on the field of application and surface properties addressed, also lower cutting speeds could be useful. Since it is possible to achieve compressive residual stresses for cutting speeds up to 100 m/min, especially in cases in which a low surface roughness value is not required.

Diamond smoothing acts as an effective final surface treatment and, by applying moderate smoothing forces (100 –150 N) simultaneously minimizes surface roughness depth, induces high compressive residual stresses, increases surface microhardness, and further refines crystallite size, indicating a combination of surface flattening and near-surface plastic deformation.

Overall, this work establishes a mechanistic framework linking machining parameters to surface geometry, residual stress state, and microstructural refinement in pure nickel. By identifying parameter regimes that promote smooth surfaces with compressive residual stresses and refined surface layers, the results provide a grounded basis for designing surface preparation strategies in applications where surface state is critical, such as diffusion bonding.

Although this study examines the effects of turning and smoothing parameters on surface properties, further work is needed to directly correlate these surface characteristics with diffusion bonding strength and quality. In particular, further studies are needed to examine the effect of the pre-machined surface state on the outcomes of diamond smoothing. Moreover, analyses of chip formation, tool wear mechanisms, and cutting temperature are needed to better understand the effects beyond kinematic roughness.

## Data Availability

The datasets generated and analyzed during the current study are available from the corresponding author on reasonable request.
